# 1431. Evaluating Physician Decision Making in Inpatient Antibiotic Prescription for Suspected Urinary Tract Infection

**DOI:** 10.1093/ofid/ofab466.1623

**Published:** 2021-12-04

**Authors:** Celine Arar, Valerie Reyna, Marshall J Glesby, Justin J Choi

**Affiliations:** 1 Weill Cornell Medicine, New York, New York; 2 Cornell University, Ithaca, New York; 3 Weill Cornell Medicine of Cornell University, New York, NY

## Abstract

**Background:**

Physicians are constantly asked to evaluate inpatients for possible antibiotic treatment. As part of antibiotic stewardship it is imperative to understand the decision-making process behind a physician’s choice to prescribe antibiotics appropriately in an inpatient setting. Fuzzy Trace Theory (FTT) suggests that physicians use one of two methods in medical decision making; verbatim, employing a comprehensive risk benefit analysis, and gist, considering a bottom line analysis.

**Methods:**

Seventy-six hospitalists at Weill Cornell Medicine in Manhattan, New York received a survey with two reminders to evaluate their decision-making process. Five basic demographic questions regarding participant gender, race, background, age, and years in practice were asked. A clinical vignette describing an inpatient with a possible urinary tract infection (UTI) was followed with statements framing hypothetical antibiotic prescription. A seven point Likert scale with response choices from Strongly Disagree scored as one to Strongly Agree scored as seven was used to assess degree of participant agreement with each statement. Questions were presented in a random order to eliminate possible effects of questions clusters or question order.

**Results:**

Twenty-six hospitalists completed the survey. Consistent with previous literature, the hospitalists surveyed displayed a gist interpretation of the risks and benefits of antibiotics, with a mean Likert scale score of 5.54 agreeing that there are benefits to antibiotic prescription, and a mean Likert scale score of 6.04, agreeing that there are risks to antibiotic prescription. . However, the clinicians surveyed ultimately found antibiotics to be a necessary risk given the possible benefit of improving patient health. The hospitalists surveyed also did not view antibiotic prescription to be a product of pressure from patient families, agreeing by a mean Likert scale score of 5.08 that the patient’s family will trust their physician to prescribe antibiotics if needed.

**Conclusion:**

These findings suggest that physician education to reduce overprescribing of antibiotics should underscore possible antibiotic risk, despite potential benefit.

**Disclosures:**

**Marshall J. Glesby, MD**, **Enzychem** (Consultant)**Gilead** (Grant/Research Support)**ReAlta Life Sciences** (Consultant)**Regeneron** (Consultant, Grant/Research Support)**Sobi** (Consultant)**Springer** (Other Financial or Material Support, Royalties)**UpToDate** (Other Financial or Material Support, Royalties) **Justin J. Choi, MD**, **Allergan** (Consultant, Grant/Research Support)**Roche** (Consultant, Grant/Research Support)

